# Transition to motherhood in type 1 diabetes: design of the pregnancy and postnatal well-being in transition questionnaires

**DOI:** 10.1186/1471-2393-13-54

**Published:** 2013-02-27

**Authors:** Bodil Rasmussen, Trisha Dunning, Christel Hendrieckx, Mari Botti, Jane Speight

**Affiliations:** 1 Deakin University, School of Nursing and Midwifery, 221 Burwood Highway, Burwood, Victoria 3125, Australia; 2 Deakin University- Barwon Health, Waterfront, PO Box 281, Geelong, Victoria 3220, Australia; 3 Australian Centre for Behavioural Research in Diabetes, Diabetes Australia-Vic, 570 Elizabeth Street, Melbourne 3000, Australia; 4 Centre for Mental Health and Well-being Research, School of Psychology, Deakin University, 221 Burwood Highway, Burwood, Victoria 3125, Australia; 5 AHP Research, 16 Walden Way, Hornchurch, UK; 6 Deakin University-Epworth HealthCare, Centre for Clinical Research Nursing, 89 Bridge Road, Richmond, Victoria 3121, Australia

**Keywords:** T1DM (type 1 diabetes), Pregnancy, Postnatal, Questionnaire, Transitions, Self-management, Social support, Psychological well-being

## Abstract

**Background:**

Life transitions are associated with high levels of stress affecting health behaviours among people with Type 1 diabetes. Transition to motherhood is a major transition with potential complications accelerated by pregnancy with risks of adverse childbirth outcomes and added anxiety and worries about pregnancy outcomes. Further, preparing and going through pregnancy requires vigilant attention to a diabetes management regimen and detailed planning of everyday activities with added stress on women. Psychological and social well-being during and after pregnancy are integral for good pregnancy outcomes for both mother and baby. The aim of this study is to establish the face and content validity of two novel measures assessing the well-being of women with type 1 diabetes in their transition to motherhood, 1) during pregnancy and 2) during the postnatal period.

**Methods:**

The approach to the development of the Pregnancy and Postnatal Well-being in T1DM Transition questionnaires was based on a four-stage pre-testing process; systematic overview of literature, items development, piloting testing of questionnaire and refinement of questionnaire. The questionnaire was reviewed at every stage by expert clinicians, researchers and representatives from consumer groups. The cognitive debriefing approach confirmed relevance of issues and identified additional items.

**Results:**

The literature review and interviews identified three main areas impacting on the women’s postnatal self-management; (1) psychological well-being; (2) social environment, (3) physical (maternal and fetal) well-being. The cognitive debriefing in pilot testing of the questionnaire identified that immediate postnatal period was difficult, particularly when the women were breastfeeding and felt depressed.

**Conclusions:**

The questionnaires fill an important gap by systematically assessing the psychosocial needs of women with type 1 diabetes during pregnancy and in the immediate postnatal period. The questionnaires can be used in larger data collection to establish psychometric properties. The questionnaires potentially play a key role in prospective research to determine the self-management and psychological needs of women with type 1 diabetes transitioning to motherhood and to evaluate health education interventions.

## Background

Managing type 1 diabetes (T1DM) has a major effect on the individual’s lifestyle in the short and long term, due to the daily demands of self-monitoring, taking insulin and managing blood glucose. People with T1DM need to make complex decisions, not least during transitional periods, e.g. going to college, entering pregnancy or parenthood [1,2]. Life transitions are peak times of change, which increase stress and affect problem-solving and coping abilities [2-5]. The added stress during transitions often makes managing blood glucose levels (BGLs) particularly difficult for young women with T1DM, who describe the experience as ‘being in the grip of blood glucose’ [5].

Pre-existing diabetes in pregnancy affects less than one per cent of pregnancies in Australia, but the high risk and potential for serious and long-lasting outcomes make it an important issue for healthcare providers [6]. Pregnancy in women with T1DM requires careful preconception planning and management throughout gestation [7]. Women with T1DM are at higher risk of adverse pregnancy outcomes and are more likely to have a stillbirth, pre-term induced labour and birth, caesarean section, hypertension and longer stay in hospital than women with gestational diabetes (GDM) or without diabetes [8]. Babies of mothers with T1DM typically have, higher birth weight, lower Apgar scores, and are more likely to require high-level resuscitation, admission to special care nursery/neonatal intensive care unit, and longer stay in hospital than babies of mothers with GDM or without diabetes [6]. Furthermore, mothers with T1DM have higher rates of caesarean section, hypertension and pre-term birth than mothers with type 2 diabetes [6].

Coupling the increased risk of diabetes-related complications accelerated by pregnancy with risk of adverse childbirth outcomes, pregnancy is typically a time of heightened anxiety and stress for the woman [9] placing her in a uniquely challenging position. Preparing for and going through pregnancy thus requires close attention to a strict diabetes management regimen and detailed planning of everyday activities. Daily life is characterised by exaggerated feelings of responsibility and perceived demands from the baby, creating constant worry, self-blame, guilt and fear of being a ‘burden’ to others and pressure to provide the best conditions to enable delivery of a healthy baby [10-12].

The challenge of maintaining glycaemic stability continues into the postnatal phase. Women typically perceive a strong need for monitoring and controlling their BGLs in order not to jeopardise their capacity to care for their newborn infant [13]. The women fear that unexpected hypoglycaemic events would impair their ability to care for their child [5,13], in particular when breastfeeding [13]. The women’s need for support increases in this phase as they feel abandoned by or disconnected from health professionals [11], which underlines the need for extra support during the postnatal period.

The social environment plays an integral role in women’s perceptions of stress and sense of control of both diabetes and their transition to motherhood [1,5]. In spite of evidence that stress and social support may impact on health behaviours and outcomes of diabetes pregnancy [9,14]; few studies have examined the effect of social support immediately after pregnancy in women with T1DM.

Psychological and social well-being during and after pregnancy are integral for good pregnancy outcomes for both mother and baby [15,16], yet there is currently no single measure that specifically captures the complexities of both diabetes- and pregnancy/postnatal-related psychological and social well-being in this group. Thus, the aim of this study was to design two related questionnaires that identify the facilitators and barriers to self-care and psychosocial well-being among women with T1DM in the transition to motherhood, during and after pregnancy.

## Methods

**Figure 1:**
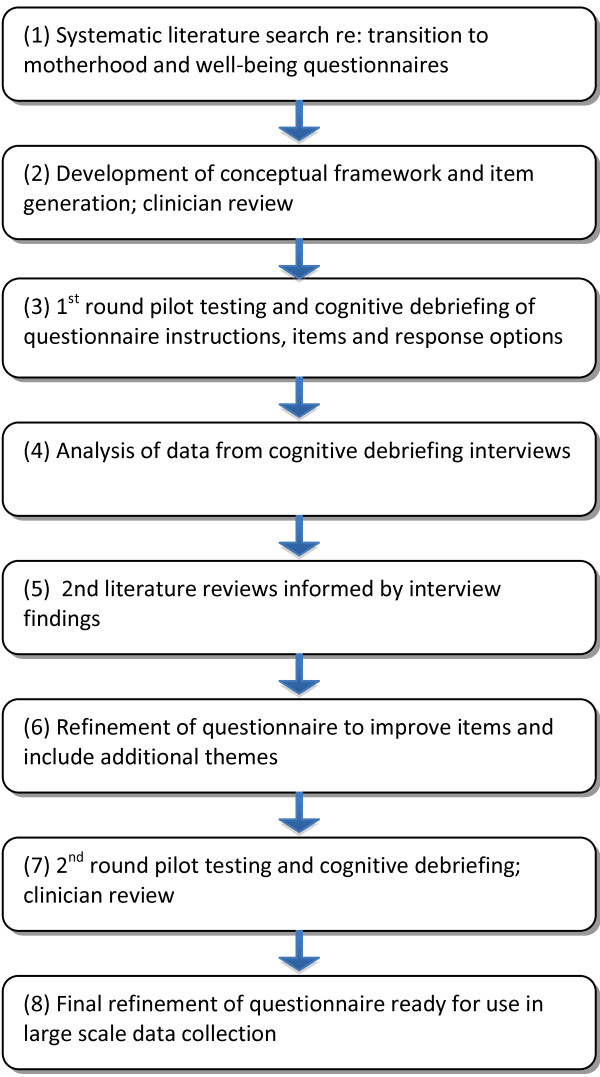
Questionnaire development process.

We developed two separate questionnaires for pregnancy and the postnatal period to maximise each the face and content validity of each the measure. The development of the questionnaires was based on a detailed and iterative process of literature reviews, item generation, expert review, piloting testing and cognitive debriefing of the draft questionnaires, and item refinement (see Figure 1). Ethics approval was granted by Deakin University Human Ethics Committee.

### Literature review

A literature search was conducted to identify questionnaires relevant to well-being, diabetes-specific well-being and pregnancy-related well-being. Many general well-being questionnaires were identified that have been used in studies of pregnancy and the postnatal period [17-20]. Similarly, several diabetes-specific measures were identified [9,16,21]. However, none covered the well-being and social support needs of women with T1DM specifically during or after pregnancy.

Consequently, we undertook a systematic search of the literature to identify the needs and experiences prior, during and after pregnancy of women with T1DM and identified 15 studies which we describe in detail in a narrative review elsewhere [22].

The review indicated that women with T1DM experienced a variety of psychosocial issues in their transition to motherhood: increased levels of anxiety, diabetes-related distress, guilt, a sense of disconnectedness from health professionals, and a focus on medicalization of pregnancy rather than the positive transition to motherhood. A trusting relationship with health professionals, sharing experiences with other women with T1DM, active social support, shared decision and responsibilities for diabetes management assisted the women to make a positive transition.

There was a high level of diversity between the aims of the included studies but three common key themes of women’s experiences with diabetes in pregnancy and in the postnatal period were identified: psychological well-being, social environment and physical (maternal and fetal) well-being.

### Development of conceptual framework and item generation

Based on the literature review, a conceptual framework was developed for the questionnaire design, incorporating three distinct but related themes (or proposed subscales): social environment, concerns regarding physical well-being and psychological well-being. These themes were then were elucidated into specific concepts and items developed relating to the pregnancy and postnatal periods (see Table 1).

**Table 1:** Conceptual framework and final item wording

**Theme**	**Concept**	**Final item wording**	
		**Pregnancy version**	**Postnatal version**
**Social environment**	General social support	I feel well supported during my pregnancy.	I feel well supported during the first weeks after giving birth.
	People – understand challenges	I feel that people around me understand the challenges of having diabetes and being pregnant.	I feel that people around me understand the challenges of having diabetes and caring for a baby.
	People – support emotional	I feel emotionally supported by my partner since I became pregnant.	I feel emotionally supported by my partner since my baby has arrived.
		I feel emotionally supported by my family (e.g. parents, in-laws, brothers, sisters).	I feel emotionally supported by my family (e.g. parents, in-laws, brothers, sisters).
	Partner – support practicalities	I feel supported by my partner with the practicalities of being pregnant.	I feel supported by my partner with the practicalities of caring for our baby.
		I feel supported by my family (e.g. parents, in-laws, brothers, sisters) with the practicalities of being pregnant.	I feel supported by my family (e.g. parents, in-laws, brothers, sisters) with the practicalities of caring for my baby.
	Health professionals support	My health professionals help me to understand what I want to know.	My health professionals helped me to understand what I want to know.
		My health professionals prepared me for what to expect whilst being pregnant.	My health professionals prepared me for what to expect after giving birth.
		My health professionals equipped me with the skills needed to manage my diabetes while being pregnant.	My health professionals equipped me with the skills needed to manage my diabetes after giving birth
		I feel supported by my health professionals.	I feel supported by my health professionals.
		I can always talk openly with my health professionals about how I feel.	I can always talk openly with my health professionals about how I feel.
		My health professionals only give me information about my unborn baby’s health when I ask questions.	My health professionals only give me information about my baby’s health when I ask questions.
		My health professionals always discuss my care plan with me.	My health professionals always discuss my care plan with me.
	Information	I have enough information about caring for a unborn baby whilst having diabetes*	I have enough information about caring for a baby whilst having diabetes*
	Family interactions	My family claims they know what is best for my diabetes.	My family claims they know what is best for my diabetes.
		My family think they know what is best for my unborn baby.	My family think they know what is best for my baby.
		My friends think they know what is best for my diabetes.	My friends think they know what is best for my diabetes.
		My friends think they know what is best for my unborn baby.	My friends think they know what is best for my baby.
**Concerns related to physical (maternal and fetal) wellbeing**	Anxiety – managing BG levels	I feel anxious managing my diabetes because my blood glucose levels have changed since becoming pregnant.	I worry about dropping my baby when I have a hypo^*^
		I worry more about low blood glucose levels now I am pregnant.	I worry more about low blood glucose levels now thatI have to take care of a baby.
	Anxiety – developing complications	I worry more about developing new diabetes complications since I became pregnant.	I worry more about developing new diabetes complications since I became a mother.
		I worry about my unborn baby developing diabetes.	I worry about my baby developing diabetes
	Awareness - diabetes	Being pregnant made me more aware about the importance of looking after my diabetes.	Being a mother has made me more aware about looking after my diabetes.
		My unborn baby’s needs always come before my diabetes care needs.	My baby’s needs always come before my diabetes care needs.
		Being pregnant makes me realise my own health is very important.	Having a baby makes me realise my own health is very important.
	Balancing Diabetes and pregnancy/new baby	Balancing the needs of my diabetes care and my unborn baby’s needs is a real challenge.	Balancing the needs of my diabetes care and my baby’s needs is a real challenge.
		I find it easier to prioritise my long term health goals now I am pregnant.	I find it easier to prioritise my long term health goals now I am a mother.
	Breastfeeding^*^		I received adequate information about how breastfeeding impacts on blood glucose levels.*
			My health professionals explained how breastfeeding could affect my blood glucose levels.*
			My health professionals explained how to manage my blood glucose levels when breast feeding.*
**Psychological well-being**	Optimistic – healthy baby	I feel optimistic about my baby’s future health.	I feel optimistic about my baby’s future health.
	Optimistic – not developing complications	I feel optimistic about my personal risk of developing diabetes complications.	I feel optimistic about my personal risk of developing diabetes complications
	Sense of achievement	Managing my diabetes whilst being pregnant gives me a sense of achievement	Managing my diabetes whilst caring for my baby gives me a sense of achievement.
	Coping – with baby and diabetes	I am coping well with looking after my pregnancy and diabetes.	I am coping well with looking after both my baby and diabetes.
	Competence*	I feel competent overall that I can manage whatever being pregnant involves*.	I feel competent overall in caring for my baby*.
		I feel I can manage my diabetes no matter what*.	I feel I can manage my diabetes no matter what*.
	Anxiety about new role	I feel anxious about my diabetes management since becoming pregnant.	I feel anxious about my diabetes management since becoming a mother.
	Judgemental attitudes – others	I worry that others judge my ability to care for my unborn baby because I have diabetes.	I worry that health professionals judge my ability to care for my baby because I have diabetes.
	Guilt feelings	I feel guilty knowing diabetes might affect my unborn baby’s health.	I feel guilty knowing that diabetes might affect my baby’s health.
		I feel guilty about the affect my diabetes has on family and friends now I am pregnant.	I feel guilty about the affect my diabetes has on family and friends now I have a baby.
	Motivation	Being pregnant motivates me to look after my diabetes.	Having a baby motivates me to look after my diabetes.
	Sense of loneliness	I feel alone caring for my pregnancy.	I feel alone caring for my baby.
	Depression	I have little interest or pleasure in doing things since I became pregnant.	I have little interest or pleasure in doing things since I gave birth.
		I have trouble making any decisions now I am pregnant.	I have trouble making any decisions now I am a mother.
	Confidence	I feel confident I can do everything I need to do for me and my unborn baby.	I feel confident I can do everything I need to do for me and my baby.
Miscellaneous	Financial impact of entering motherhood*	Financial costs are a barrier to managing my diabetes now I am pregnant.	Financial costs are a barrier to managing my diabetes now I am a mother.
		The financial costs of managing my diabetes worry me more now I am pregnant.	The financial costs of managing my diabetes worry me more now I am a mother.
	Internet*	The internet is the most common way I communicate with other pregnant women with diabetes.*	The internet is the most common way I communicate with other new mothers with diabetes.*

*indicate items added after interviews.

Item generation was based on information derived from the literature review and previous qualitative research [1,5] and each item was discussed for clarity and relevance. The first full draft 45-item questionnaires were then reviewed by an expert panel including researchers and clinicians (representing nursing, psychology and endocrinology) and representatives from two consumer groups (The Type 1 Diabetes Network, Diabetes Australia – Vic). A five point Likert–scale response (strongly disagree to strongly agree) was applied to each of the items.

### Pilot testing and cognitive debriefing of the questionnaires

The questionnaires were pilot tested with eight women with T1DM, recruited via a local support group for young people with T1DM. Participants completed the questionnaires unassisted and then took part in face-to-face cognitive debriefing (CD) interviews (conducted by BR), with the aim of establishing the face and content validity of the items, i.e. that the instrument is measuring what it claims to be measuring. CD interviews involve incorporating standardised follow-up questions to understand how the intended population interprets the instructions, questions and response options, to enable improved clarity and appropriateness of wording (identifying any ambiguity in items and/or response options), to identify repetition and redundancy, and to identify additional items that need to be included [23].

For each questionnaire item, a series of clarifying questions were asked: “Did you have any difficulty understanding this item?”; “What does it mean to you?”; “Is the item relevant to you?”; “Are any words difficult to understand?”; “Would you use any other words?”; and “Are the response options appropriate?”. The interviews lasted 1.5 to 2 hours and were audio-recorded. Participant responses were noted in a summary grid describing the women’s comments and recommendations for changes.

### Refining the questionnaires

The summary grid was used as the basis for the discussion with the rest of the research team and was used to refine questionnaire items. After all interviews were complete, the audio recordings were analysed for comments supporting the various themes and concepts. The second version of the questionnaires was pilot tested with two additional women with T1DM and the final version again reviewed by the expert panel.

## Results

### Participants

The participants were aged 27 to 40 years and had lived with T1DM for 3 to 27 years (see Table 2). Each had been diagnosed with T1DM prior to becoming pregnant and were either pregnant at the time of interview or had given birth within the past four to 18 months.

**Table 2:** Demographic and clinical characteristics of participants

	**N (%) or median**	**Min**	**Max**
**Age (years)**	31	27	40
**Duration of diabetes (years)**	18	3	27
**Diabetes treatment**			
**- pump therapy**	4 (40%)	-	-
**- multiple daily injections**	6 (60%)	-	-
**Number of pregnancies**	1.5	1	8
**- pregnant at time of interview**	4 (40%)	-	-
**Infant under 12 months at time of interview**	5 (50%)	-	-
**Living in metropolitan/regional area**	7 (70%)/3 (30%)	-	-
**Living with partner**	9 (90%)	-	-
**Education level**			
**- tertiary**	7 (70%)	-	-
**- TAFE**	1 (10%)	-	-
**- secondary school**	2 (20%)	-	-

### Cognitive debriefing

Overall, the themes of the conceptual framework were endorsed by the women and the questionnaire was regarded as highly relevant. Most items were easily understood by the women and did not require substantive content changes.

#### Psychological well-being

To capture the notion of psychological well-being, 15 items were developed about perceived competence as a mother (to be), perceived control, coping mechanisms, optimism and future planning, and other factors contributing to overall psychological well-being. Positive mental health and well-being was endorsed by all participants as an overarching issue, as it was important for them not to focus overly on the diabetes-related risks and medical aspects of their pregnancy and postpartum period but rather on the normal and joyous aspects of becoming a mother.

Based on the interviews the psychological impact of diabetes during pregnancy was particularly related to feeling guilty about the impact diabetes had on the women’s partners, families and friends; and the change of identity in the context of perceived self-worth and increased anxiety, which was associated with the women’s ability to cope with diabetes and pregnancy:

*I am normally not an anxious or depressed person but during my pregnancy I am. It changes at different stages in the pregnancy too* (Int D)

There was consensus among the interviewed women that it was difficult to retain positive mental well-being during and after the pregnancy. In particular, women who had given birth felt they were unprepared for the immediate period at home with a new baby. Four of the women they had were postnatal depressed, which had a major impact on their diabetes management and the way they cared for their babies. An additional literature review was conducted regarding postnatal depression among women with T1DM (see below) to determine if the newly developed items were sufficient to capture those most at risk.

Women also highlighted the financial impact of having diabetes and being pregnant. Items were developed to explore women’s beliefs about the cost of extra scans, increased visits to healthcare specialists, transport and time away from work, all added stress and worries to the women and their families.

#### Social environment

Social environment in the current study refers to the amount or level of support women perceived receiving during their pregnancy and in the immediate postnatal period. Eighteen items related to support from partners, other family members (in particular, mothers), health professionals, information about what to expect, contact with other women with T1DM and online communications.

The interviewees considered social support an integral facilitator (or barrier if missing) of good outcomes. While most women felt well supported by healthcare professionals during pregnancy, seven of the ten highlighted the lack of support from healthcare providers in the immediate postnatal period. They felt ill prepared for how to adapt to their new situation:

*Once I was postnatal they [the health professionals] all ditched me. When the baby is born it is all over [referring to services and health professionals]. I was left behind after giving birth. You can feel helpless with a new baby. I have no family around me and my husband is working all day. It is very hard and no health professional seems to care* (Int B)

The interview feedback also indicated that the social support, knowledge and skills gained by using websites and e-mails had a positive impact on the women’s self-perceptions and helped them to feel more confident about themselves and their diabetes management. Two items were added about competence and one item specifically related to use of Internet.

*Internet was my best support. There is very little support as soon as you have delivered the baby. Internet was good to connect with other women with diabetes. I google the question and get the answers* (Int D)

Five items related to need for information, accessing information, and perception of being prepared for the changes in diabetes management. The women reported feeling ill-informed about how breastfeeding would impact on their diabetes management. It was apparent that breastfeeding was a particularly complex issue for women in the postnatal period. An additional literature review focusing on breastfeeding issues for women with diabetes was conducted (see below) and three items were added to the postnatal questionnaire, related to knowledge and support about breastfeeding’s impact on diabetes management and breastfeeding support mechanisms:

*I remember I had just injected [insulin] and the baby started to cry. I had to breastfeed and suddenly an hour was gone. It was very difficult and no-one prepares you for this difficulty. I am not sure that women [with T1DM] know anything about how breastfeeding influence blood glucose levels* (Int A)

#### Concerns related to physical (maternal and fetal) well-being

The transition to motherhood is particularly difficult because pregnancy requires strict diabetes management, vigilant planning of everyday activities of constant awareness of risks to one’s own and the (unborn) child’s health. Nine items plus 3 specific related to breastfeeding were developed to cover issues such as fear of hypoglycaemia (hypos) and hyperglycaemia (which both increase during pregnancy), concerns for the baby’s health, fears of being stigmatised or judged as an incompetent mother by others as well as physical harm to themselves or their baby during hypos or accidents. Participants endorsed the items favourably, with one participant commenting:

*You have [knowledge about] the hypo bit but not the hyper which is you can fall over with a hypo but with a hyper you can cause birth defects* (Int E)

Pregnancy symptoms such as morning sickness were also an issue that the majority of the women reported as being challenging:

*Morning sickness was really hard to cope with when you have diabetes [because it is] difficult to manage when you just injected [insulin] and then start to vomit* [Int B]

The women also highlighted the financial impact of pregnancy and becoming a mother. They indicated that the extra costs related to increased visits to specialists, transport expenses, costs associated with ultrasound scans as well as loss of income and extra costs associated with being pregnant or having a baby. Social and family condition played a big role in the women’s perception of financial impact on diabetes management. Two new items were developed to assess barriers to managing diabetes and how much financial impact worried them.

#### Miscellaneous

The CD interviews also highlighted that certain items were not applicable to everyone, e.g. “internet chat rooms are the only way I communicate with other women with T1DM” was not relevant if the women did not have computer access. Subsequently, ‘not applicable’ response options were included where necessary.

### Further literature reviews

A search of the breastfeeding literature review identified a total of nineteen articles about breastfeeding and diabetes, eight of which specifically focused on breastfeeding among women with T1DM. The majority of articles concerned the complications in children of mothers with T1DM. One finding of significance was that long-term breastfeeding (more than 6 months) was significantly related to higher education level [24]. T1DM was not an independent risk factor for shorter duration of breastfeeding [25]. However, in line with previous studies in women without diabetes, breastfeeding among women with T1DM was less likely among those who had caesarean sections, early delivery, were younger and had a lower education level [26]. This review clearly indicated that initiation of early breastfeeding postpartum is vital for ongoing successful breastfeeding among women with T1DM. The women in the current study highlighted that the early stage of postpartum was the most difficult and instrumental (practical) support was integral to duration of breastfeeding.

The most commonly used validated measures for postnatal depression are the Beck Post Natal Depression Inventory-Revised (PDPI-R) [27,28] and the Edinburgh Postnatal Depression Scale (EPDS) [29]. These instruments have been used to assess postnatal depression in women with gestational diabetes [30], however we could not identify published studies in women with T1DM. In contrast to the large numbers of papers reporting on the association between diabetes and depression, there is a lack of research investigating postnatal depression in women with T1DM. Some studies have used a generic depression scale [31-33]. For example, Dalfra et al. [33] indicated that women with T1DM as well as women with gestational diabetes had higher scores on the Centre for Epidemiological Studies – Depression (CES-D) postnatally compared to the 3^rd^ trimester of pregnancy, and also compared to women without diabetes at both time points. Others have reported on samples of women with pre-existing diabetes not showing any difference in depression scores, as measured by the revised Beck Depression Inventory (BDI-R), compared to women with gestational diabetes or women without diabetes [34]. These inconsistent findings could be due to differences in the measures used. For example, it has been suggested that the CES-D measures distress rather than depression [35]. Several items of our newly developed postnatal questionnaire compliment the 13 items of the PDRI-R suggesting our measure could be used alongside the PDPI-R to enable detection of diabetes-specific as well as generic psychological distress in the postnatal period.

## Discussion

Based on literature reviews, a previous qualitative study and cognitive debriefing interviews, and in consultation with an expert panel, a 45-item (pregnancy) and a 49-item (postnatal) self-report questionnaire were designed, enabling assessment of important psychosocial issues for women with T1DM during pregnancy and in the immediate postnatal period. Rigorous design of the questionnaires was the necessary first stage in a program of research to enable systematic assessment of facilitators and barriers to self-management and psychological well-being of women with T1DM in the transition to motherhood.

The results of the study indicate that the questionnaires have good face and content validity, which was established in several ways. First, the literature review and previous qualitative research informed the questionnaires’ conceptual framework and item generation. Second, during the cognitive debriefing interviews, the participating women judged the questionnaire items to be relevant as well as having sufficient clarity and readability. They identified additional areas for inclusion, for which items were developed and piloted in a second round. Third, the questionnaire items were assessed to be relevant and appropriate by the research team and expert panel, comprising internationally recognised researchers and clinicians with expertise in diabetes education, nursing, psychology and endocrinology, as well as consumer representatives.

An overview of the final questionnaires endorses the fact that life transitions are personal experiences [31]. Transitions are diverse and complex, may involve uncertainty, can be sequential or simultaneous, and often occur in multiples that compound the effect of transitions [5,36]. Transitions involve a change in health status, role relations, expectations, or abilities [5,36,37].

Psychological wellbeing is integral to the questionnaires because the concept is associated with one's ability to express or release one’s inner feelings [38,39]. Psychological well-being is a multifaceted concept which implies an ease with oneself and around others and determines one's ability to effectively and successfully manage challenges [38]. Ruff [38] identified six areas of psychological well-being, which are now more commonly referred to as positive well-being, reflecting the focus on the affirmative nature of the constructs: autonomy, environmental mastery, personal growth, positive relations with others, purpose in life, and self-acceptance. Those concepts have been identified as relevant in diabetes care [40-42], and in the current study, items were developed to assess women’s abilities to cope, their sense of optimism and beliefs in personal control over the combined demands of T1DM and pregnancy.

Women with T1DM face several specific challenges during pregnancy, childbirth and in the postpartum period. In particular, women in this study highlighted their unpreparedness for the challenges of breastfeeding and maintaining optimal blood glucose levels to ensure that neither they nor their baby were at risk due to hypoglycaemia. They lacked knowledge, skills and support from health professionals. It is therefore of vital importance to consider their need for specific support in early postpartum [13]. More research about the critical postnatal period is needed among healthcare providers and experiences need to be shared both ways between the women and their healthcare providers. The postnatal version of the questionnaire will enable assessment of the needs of women with T1DM in the postnatal period. ‘The sample size of the current study did not allow for fully exploration of any parity-related differences in women responses, e.g. anxiety levels among multiparous women could either be elevated or diminished depending on previous experience. A large-scale study would be able to better identify if support and information needs differ between primiparous and multiparous women with T1DM’.

For all women in the postpartum period (regardless of a diabetes diagnosis), it is important that early signs of postnatal depression are detected and managed appropriately. Postpartum depression is a crippling mood disorder, historically neglected in healthcare, leaving mothers to suffer in fear, confusion, and silence [26]. Undiagnosed, it can adversely affect the mother-infant relationship and lead to long-term emotional problems for the child [26]. Studies indicate that women with pre-existing diabetes experience greater anxiety and depressive moods [21,33,42], are more distressed [43] and report lower mental health [9] compared to pregnant women without diabetes. They also report more intense pregnancy-related negative feelings and fewer positive emotions than pregnant women without diabetes. In the first large-scale data collection using the newly developed postnatal questionnaire, we will also include the Postpartum Depression Predictors Inventory–Revised questionnaire (PDPI-R) [27,28]. Our intention will be to identify women with or at risk of postpartum depression and, as a secondary objective, to determine whether the newly developed questionnaires are capable of detecting those most at risk.

This study also identified that appropriate and timely information throughout the transition to motherhood was lacking and that the internet and web-based information/support plays an important role for women with T1DM during time. According to Sparud-Lundin et al. [15], a high proportion of women with T1DM seek diabetes-related information on the internet, especially before, during, and after pregnancy. In an Australian study, Rasmussen et al. [5] found that websites and e-mails had a positive impact on the self-perceptions, self-confidence and diabetes management of young women with T1DM by making it easier to access information. In addition, it reduced their sense of isolation and informed them about different approaches to health services [5]. Sparud-Lundin et al’s [15] study highlighted the importance of further developing effective web-based support that contains reliable information, interactive support and enables social networking for this population.

### Limitations

The sample size was small and the educational level of participants was high, with 70% tertiary educated. However, the combination of the previous qualitative data, expert reviews and cognitive debriefing interviews justify that the items and depth of data were acceptable for the aim of the study. The cognitive debriefing form focussed the interviews on the depth and breadth of each theme, also enabling participants to indicate where new items were needed or existing items needed to be modified. The youngest participant was 27 years of age, which might be a limitation in that younger women’s experiences, psychosocial needs and coping mechanisms may differ from those of older women. However, the women at the age of 27 years and above were able to compare more life transitions and, therefore, be more specific about their needs during their transition to the motherhood.

## Conclusion

Both of the newly developed pregnancy and postnatal questionnaires fill an important gap by enabling women with T1DM to consider specific facilitators and barriers related to social support, their well-being and specific diabetes management concerns in their transition to motherhood. Efforts were made to use everyday language to ensure the items were engaging and meaningful for this group of women. The iterative methodology resulted in improvements to the instructions and item wording so that the final questionnaires are now easily understood and highly relevant. This detailed and iterative process has established the face and content validity of the pregnancy and postnatal versions of two questionnaires concerned specifically with the well-being of women with T1DM as they transition to motherhood. Both versions are now ready for inclusion in large-scale data collections to enable full psychometric properties to be established.

Notwithstanding the limitations described above, and the need to now establish their psychometric properties the newly designed questionnaires have the potential for informing and evaluating a psycho-educational intervention designed to better support women with T1DM as they transition to motherhood and to improve communication between women and healthcare providers. The questionnaires can assist healthcare professionals to better judge the timing and content of information needed. This is critical as the women’s needs change during the different phases in transitional period to motherhood.

## Consent

Written informed consent was obtained from the patients for publications of this report and any accompanying images.

## Abbreviations

T1DM: Type 1 Diabetes.

## Competing interests

The authors declare that they have no competing interests.

## Authors’ contributions

BR collected data, performed the data analysis and drafted the manuscript. TD provided advice throughout the study, commented and assisted in editing the manuscript, CH provided substantial advice throughout the study, commented on manuscript, MB assisted in conceptualising the study and provided advice, JS provided substantial advice on the design, provided advice throughout the study and commented and edited the manuscript. All authors have approved the final manuscript.

## Pre-publication history

The pre-publication history for this paper can be accessed here:

http://www.biomedcentral.com/1471-2393/13/54/prepub

## Access to the questionnaires

For further information and permission to use the questionnaires, please contact first author Associate Professor Bodil Rasmussen, bodilr@deakin.edu.au.
